# Agmatine coumaroyltransferase gene on the short arm of chromosome 2H is involved in hordatine biosynthesis in barley

**DOI:** 10.5511/plantbiotechnology.25.1209a

**Published:** 2026-03-25

**Authors:** Naoki Ube, Taiji Nomura

**Affiliations:** 1 Biotechnology Research Center and Department of Biotechnology, Toyama Prefectural University, 5180 Kurokawa, Imizu, Toyama 939-0398, Japan

**Keywords:** agmatine coumaroyltransferase, barley, hordatine, hydroxycinnamic acid amide, *p*-coumaroylagmatine

## Abstract

Plants produce dimerized phenolic compounds as secondary metabolites. Hordatine A (HA), a dehydrodimer of *p*-coumaroylagmatine (*p*CA), is an antifungal compound that accumulates substantially in young barley (*Hordeum vulgare*) seedlings. The first committed step of the HA biosynthetic pathway is the formation of *p*CA via condensation of *p*-coumaroyl-CoA and agmatine, which is catalyzed by agmatine coumaroyltransferase (ACT). Although two ACT-encoding genes (*HvACT-2HL1/2*) on the long arm of barley chromosome 2H (2HL) have been identified, our previous study suggested the presence of another *ACT* locus on the short arm of barley chromosome 2H (2HS). In this study, an analysis of dissection lines of wheat (*Triticum aestivum*) carrying aberrant barley 2H chromosomes detected *p*CA in wheat lines carrying the distal region of 2HS. This chromosomal region, which includes genes encoding the laccase catalyzing the last committed step of the HA biosynthetic pathway, was revealed to also contain a putative *ACT* gene (*HvACT-2HS1*), with the encoded amino acid sequence similar to that of HvACT-2HL1 (46% sequence identity). Changes in *HvACT-2HS1* transcript levels were in accordance with those in the *p*CA-forming enzymatic activity and the *p*CA level in barley seedlings. Additionally, recombinant HvACT-2HS1 heterologously expressed in *Escherichia coli* had *p*CA-forming enzymatic activity, with high specificity for agmatine as the acyl acceptor. Moreover, a phylogenetic analysis indicated that *HvACT-2HS1* is not a paralog of *HvACT-2HL1/2*. These results suggest that *HvACT-2HS1* and *HvACT-2HL1/2*, which originated from different ancestral genes, jointly mediate the formation of *p*CA for HA biosynthesis in barley.

## Introduction

Hordatines are optically active secondary metabolites in cultivated and wild barley (*Hordeum vulgare* ssp. *vulgare* and *H. vulgare* ssp. *spontaneum*) (Stoessl 1966; Ube et al. 2017). Hordatines A and C (HA and HC) are homodimers of *p*-coumaroylagmatine (*p*CA) and feruloylagmatine (FA), respectively, whereas hordatine B (HB) is a heterodimer of *p*CA and FA (Gorzolka et al. 2014; Stoessl 1966; Ube et al. 2017). Hordatines also exist as glycosides, including HA and HB glucosides and the HA maltoside, which were isolated from barley seedlings and seeds (Kageyama et al. 2011; Kohyama and Ono 2013). The results of mass spectroscopic analyses of the extracts of barley seedlings and seeds suggest that there are other minor hordatines (Gorzolka et al. 2014; Pihlava 2014). Hordatines have strong antifungal effects and accumulate substantially in young seedlings (Stoessl 1967; Stoessl and Unwin 1970). Thus, they are believed to help protect vulnerable seedlings from pathogen infections. In addition, hordatines contribute to the astringent aftertaste (Kageyama et al. 2011) and antioxidative effects (Spreng and Hofmann 2018) of beer. HA can stimulate gastrointestinal motility by binding to muscarinic M3 receptors (Yokoo et al. 2004).

**Figure 1:**
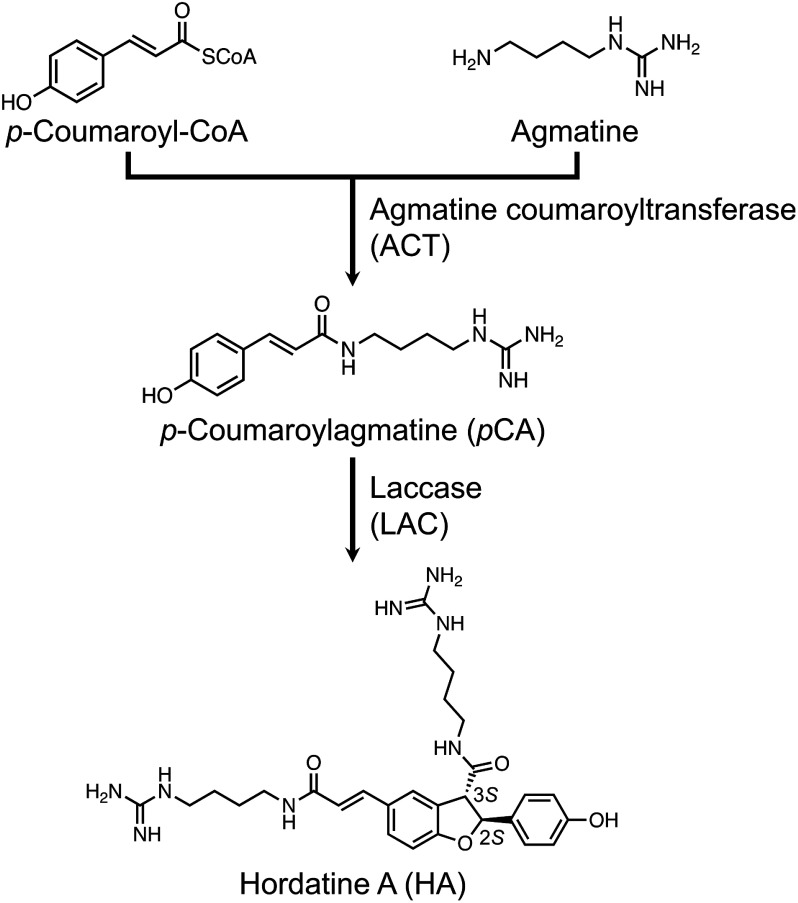
Figure 1. Hordatine A (HA) biosynthetic pathway.

HA is biosynthesized via two enzymatic reactions involving *p*-coumaroyl-CoA and agmatine (Figure 1). In the first committed step, *p*CA is formed by the condensation of *p*-coumaroyl-CoA and agmatine catalyzed by agmatine coumaroyltransferase (ACT) (Bird and Smith 1981, 1983; Burhenne et al. 2003; Nomura et al. 2007, 2018). ACT is an enzyme belonging to the BAHD acyltransferase superfamily, which was named after the first four characterized members (i.e., benzylalcohol *O*-acetyltransferase, anthocyanin *O*-hydroxycinnamoyltransferase, anthranilate *N*-hydroxycinnamoyl/benzoyltransferase, and deacetylvindoline 4-*O*-acetyltransferase) (D’Auria 2006; Petersen 2016). Burhenne et al. (2003) identified the first ACT-encoding gene (GenBank accession no. AY228552) in barley seedlings. Two ACT-encoding genes (GenBank accession nos. AB334132 and AB334133, designated as *HvACT-2HL1* and *HvACT-2HL2*, respectively, in this study) were subsequently identified in a different barley cultivar (Nomura et al. 2007, 2018), with sequences that are highly similar to that of the gene identified by Burhenne et al. (2003). Another ACT-encoding gene (GenBank accession no. BAK00935, designated as *HvACT-2HL3* in this study) was identified by Yamane et al. (2021), but *HvACT-2HL3* likely does not contribute to HA biosynthesis during the seedling stage because of its low transcript levels relative to those of the other *HvACT* genes.

The second step of the HA biosynthetic pathway involves the formation of optically active HA with the 2*S* and 3*S* absolute configuration via the oxidative coupling of *p*CA (Stoessl 1966; Wakimoto et al. 2009). The involvement of peroxidase in HA biosynthesis was proposed on the basis of earlier studies in which HA was formed in vitro in a reaction mediated by a crude enzyme extract from barley seedlings or by horseradish peroxidase in the presence of H_2_O_2_, although the resulting HA was optically inactive (Negrel and Smith 1984; Smith and Best 1978; Stoessl 1966, 1967). We recently revealed that laccase mediates HA formation, while also identifying two laccase-encoding genes (*HvLAC1* and *HvLAC2*) (Ube et al. 2023). Notably, HA formed in reactions catalyzed by HvLAC1/2 is optically active.

Barley HA biosynthetic genes (*HvACT*s and *HvLAC*s) are located on chromosome 2H. More specifically, *HvACT-2HL1/2* are on the long arm of 2H (2HL), whereas *HvLAC1/2* are on the short arm of 2H (2HS) (Nomura et al. 1999, 2007; Ube et al. 2023). Nomura et al. (1999, 2007) examined wheat (*Triticum aestivum*) lines carrying barley chromosomes (wheat–barley chromosome addition lines) for the presence of HA and *p*CA; they detected HA in 2H and 2HS addition lines, suggesting that HA biosynthetic genes are located on 2HS. However, *p*CA contents increased significantly in 2H and 2HS addition lines, but also in the 2HL addition line. Considering *HvACT* genes were detected on 2HL, it was predicted that there is another ACT-encoding gene located on 2HS (Nomura et al. 2007). In the present study, we identified a specific region of barley chromosome 2HS, which is responsible for *p*CA formation, via a chemical analysis of dissection lines of wheat carrying aberrant barley 2H chromosomes. By examining the genomic sequence of the identified 2HS chromosomal region, we detected a putative ACT-encoding gene (designated as *HvACT-2HS1* in this study). *HvACT-2HS1* was heterologously expressed in *Escherichia coli* for an analysis of the enzymatic properties of the encoded protein. This study revealed HvACT-2HS1 is involved in the formation of *p*CA for HA biosynthesis in barley.

## Materials and methods

### Plant materials

Barley (*H. vulgare* ssp. *vulgare* cv. ‘Betzes’), wheat (*T. aestivum* cv. ‘Chinese Spring’, CS), and barley chromosome 2H dissection and addition lines of wheat (Islam et al. 1981; Joshi et al. 2011) were obtained from seed stocks in the Faculty of Agriculture, Tottori University, Japan, and the Faculty of Agriculture, Ryukoku University, Japan. The 2H chromosomal region in each 2H dissection line is shown in Supplementary Table S1. Barley and wheat seeds were sown on a layer of wet filter paper in a Petri dish, kept at 4°C in darkness for 1 day to stimulate germination, and incubated at 25°C with a 14-h light/10-h dark cycle for several days. The appearance of barley seedlings grown under the experimental conditions is shown in Supplementary Figure S1.

### Chemicals

*p*CA, FA, *p*-coumaroylputrescine (*p*CP), and feruloylputrescine (FP) were prepared as previously described (Nomura et al. 2013a; Ube et al. 2017). *p*-Coumaroyl-/feruloyl-CoAs were also prepared as previously described (Nomura et al. 2018). Agmatine sulfate and putrescine dihydrochloride were purchased from Tokyo Chemical Industry (Tokyo, Japan) and Nacalai Tesque (Kyoto, Japan). *N*-Cyclohexyl-2-aminoethanesulfonic acid (CHES) was purchased from Dojindo Laboratories (Kumamoto, Japan). Other common reagents used in this study were purchased from Fujifilm Wako Pure Chemical (Osaka, Japan), Tokyo Chemical Industry, and Nacalai Tesque. All reagents used in this study were guaranteed grade.

### Extraction and analysis of metabolites from barley and wheat

Metabolites were extracted from the shoots and roots of barley (48- to 120-h-old), 2H addition lines of wheat (72-h-old), and 2H dissection lines of wheat (72-h-old) by immersing them in 10 volumes of 80% (v/v) methanol for 24 h. After a centrifugation at 15,000×g for 10 min (CF15RXII, Eppendorf Himac Technologies Co., Ltd., Hitachinaka, Japan), the supernatants were analyzed using a reversed-phase HPLC system (system, LC-2010CHT, Shimadzu, Kyoto, Japan; column, Cosmosil 5C_18_-AR-II, 5 µm, 4.6×150 mm, Nacalai Tesque; solvent, 10–20% (v/v) B/(A+B) in 30 min, A: 0.1% (v/v) trifluoroacetic acid, B: acetonitrile; flow rate, 0.8 ml min^−1^; detection wavelength, 280 nm; and column temperature, 40°C).

### Selection of *HvACT-2HS1* from a genomic sequence database

The HvACT-2HL1 amino acid sequence was used as a query for a BLAST search of BARLEX (https://apex.ipk-gatersleben.de/apex/f?p=284:10 (Accessed May 29, 2025)) with barley (cv. ‘Morex’) genome assembly version Hv_IBSC_PGSB_v2 (Mascher et al. 2017). *HvACT-2HS1* was selected as a candidate gene; the encoded amino acid sequence was >70% similar to the HvACT-2HL1 amino acid sequence. *HvACT-2HS1* genomic location and expression profile data were obtained from BARLEX. Details regarding this gene are provided in Supplementary Table S2.

### Phylogenetic analysis of HvACT-2HS1 and related sequences

A phylogenetic analysis of HvACT-2HS1 was performed using reported Clade IV BAHD acyltransferase superfamily sequences, which were obtained from GenBank (https://www.ncbi.nlm.nih.gov/genbank/ (Accessed May 19, 2025)), the Rice Genome Annotation Project database (http://rice.plantbiology.msu.edu/index.shtml (Accessed Jan 24, 2025)), and BARLEX. Details regarding the sequences included in this analysis are provided in Supplementary Table S3. Amino acid sequences were aligned using ClustalW and then unrooted dendrogram was constructed according to the neighbor-joining method (with *p*-distance) using MEGA 11 (https://www.megasoftware.net/ (Accessed Jan 24, 2025)) (Tamura et al. 2021). The robustness of the branches in the dendrogram was assessed by performing a bootstrap analysis with 1,000 replicates.

### Cloning of *HvACT-2HS1* cDNA

Total RNA was purified from 72-h-old barley shoots using a NucleoSpin RNA Plant Kit (Takara Bio, Kusatsu, Japan). The purified RNA was reverse transcribed using a PrimeScript RT reagent Kit with gDNA Eraser (Takara Bio). The resulting cDNA served as a template for the amplification of the *HvACT-2HS1* cDNA sequence by PCR using KOD Plus Neo DNA polymerase (Toyobo, Osaka, Japan) and gene-specific primers (Supplementary Table S4). The PCR program was as follows: 94°C for 2 min; 35 cycles of 98°C for 10 s, 63°C for 10 s, and 68°C for 1 min. PCR products of the expected size were gel-purified using a Wizard SV Gel and PCR Clean-Up System (Promega, Madison, WI, USA) and then inserted into the *NdeI* site of the pET28a vector (Novagen, Madison, WI, USA) using an In-Fusion HD Cloning Kit (Takara Bio). The recombinant plasmid was introduced into *E. coli* DH5α cells and sequenced to verify that the obtained *HvACT-2HS1* cDNA sequence (GenBank accession no. LC877952) was identical to the corresponding sequences in the barley genome database.

### Expression and purification of recombinant HvACT-2HS1

The HvACT-2HS1/pET28a plasmid was introduced into *E. coli* strain Rosetta 2 (DE3) cells (Novagen). The resulting *E. coli* cells were cultured and *HvACT-2HS1* expression was induced as described previously (Nomura et al. 2013b). The following steps were performed at 4°C. Recombinant *E. coli* cells from a 500 ml culture were harvested by centrifugation (6,000×g, 10 min) and resuspended in 20 ml extraction buffer consisting of 50 mM HEPES-NaOH buffer (pH 7.5) and 200 mM NaCl. Cells were sonicated (30 s at 100 W; six times) and centrifuged at 15,000×g for 30 min. The supernatant was added to TALON metal affinity resin (1 ml bed volume, Clontech, Palo Alto, CA, USA), which was equilibrated with extraction buffer, and mixed gently for 60 min. The resin was washed twice with 20 ml extraction buffer, after which His-tagged recombinant HvACT-2HS1 was eluted using 5 ml elution buffer consisting of 50 mM HEPES-NaOH buffer (pH 7.5), 200 mM NaCl, and 200 mM imidazole. To exchange the buffer, the eluted enzyme solution was passed through a PD-10 column (10 mM potassium phosphate (KPi) buffer containing 10 mM NaCl and 10% (v/v) glycerol). The recombinant enzyme was digested with thrombin (3 U mg^−1^ protein, Merck, Darmstadt, Germany) for 3 days to remove the His-tag. The digested enzyme solution was passed through TALON metal affinity resin in a column, which was equilibrated with 10 mM KPi buffer containing 10 mM NaCl and 10% (v/v) glycerol. The resin was washed with 5 ml of the same buffer and then the flowthrough and wash fractions were combined and added to DEAE-Toyopearl resin in a column (1.0×0.6 cm, 0.5 ml bed volume, Cl^−^-form, Tosoh, Tokyo, Japan), which was equilibrated with the same buffer. After washing with 2.5 ml of the same buffer, the His-tag-free recombinant enzyme was eluted from the column using 20, 30, 40, 50, and 100 mM KPi buffer containing 10 mM NaCl and 10% (v/v) glycerol (2.5 ml per buffer). The 50 mM KPi fraction, which contained the His-tag-free recombinant enzyme, was stored at −30°C until used in an enzyme assay.

### Protein analysis

Protein concentrations were determined using a Protein Assay Kit (Bio-Rad, Hercules, CA, USA), with bovine serum albumin serving as the standard. The molecular mass of the denatured form of the enzyme was estimated by a 10% SDS-PAGE analysis with Molecular Weight Standards Low Range (Bio-Rad). The SDS-PAGE gel was stained with CBB R-250. The molecular mass of the native form of the enzyme was estimated by a gel-filtration analysis involving a Superdex 200 Increase column (Cytiva, Marlborough, MA, USA) and the Gel Filtration Kit HMW marker (Cytiva). Proteins were eluted using 50 mM KPi buffer containing 150 mM NaCl and 10% (v/v) glycerol at a flow rate of 0.4 ml min^−1^.

### Enzyme assay

Standard enzyme reactions were performed for 20 min at 40°C in 100 mM Tris-HCl buffer (pH 8.5) containing 100 µM *p*-coumaroyl CoA, 100 µM agmatine, and 20 µl of appropriately diluted enzyme solution (100 µl final volume). The enzyme concentration and reaction time were adjusted to ensure that the reaction proceeded linearly. After stopping the reaction by adding 10 µl 1 M HCl, the reaction solution was diluted with 90 µl methanol and then centrifuged at 15,000×g for 10 min at 4°C. The supernatant was subjected to the HPLC analysis (column, TSKgel ODS-100 V, 5 µm, 4.6×150 mm, Tosoh; solvent, 12% (v/v) acetonitrile containing 0.1% (v/v) trifluoroacetic acid; flow rate, 0.8 ml min^−1^; and detection wavelength, 280 nm).

To examine the effects of pH and temperature on enzymatic activity, enzyme reactions were performed under standard assay conditions at 40°C in 100 mM sodium acetate buffer (pH 4.0–6.0), 100 mM KPi buffer (pH 6.0–8.0), 100 mM Tris-HCl buffer (pH 7.5–9.0), and 100 mM CHES-NaOH buffer (pH 9.0–10.0) or at 15–60°C in 100 mM Tris-HCl buffer (pH 8.5). To examine the effects of pH and temperature on enzyme stability, enzyme reactions were performed under standard assay conditions after pre-incubating the enzyme for 30 min at 25°C in the above-mentioned buffers (pH 4.0–10.0) or at 15–60°C in 100 mM KPi buffer (pH 7.5).

To analyze the specificity for acyl donors, enzyme reactions were performed under standard assay conditions using 100 µM agmatine as the acyl acceptor and 100 µM *p*-coumaroyl-/feruloyl-CoAs as acyl donors. To analyze the specificity for acyl acceptors, enzyme reactions were performed under standard assay conditions using 100 µM *p*-coumaroyl-CoA as the acyl donor and 100 µM agmatine/putrescine as acyl acceptors.

To determine kinetic parameters for *p*-coumaroyl-/feruloyl-CoAs, enzyme reactions were performed under standard assay conditions using 100 µM agmatine as the fixed acyl acceptor and 1–100 µM *p*-coumaroyl-CoA or 2–50 µM feruloyl-CoA as the acyl donor. To determine kinetic parameters for agmatine/putrescine, enzyme reactions were performed under standard assay conditions using 100 µM *p*-coumaroyl-CoA as the fixed acyl donor and 2–200 µM agmatine or 1–30 mM putrescine as the acyl acceptor. Kinetic parameters were determined via non-linear fitting of the data to the Michaelis–Menten equation using SigmaPlot (version 12.5, Systat Software, San Jose, CA, USA).

To compare the kinetic parameters of HvACT-2HS1 and HvACT-2HL1 under the same reaction conditions, HvACT-2HS1-catalyzed reactions were performed using the same conditions as those used for HvACT-2HL1 (Nomura et al. 2018). Specifically, enzyme reactions were performed for 20 min at 25°C in 100 mM Tris-HCl (pH 7.5).

### Time-course analysis of *p*CA-forming enzymatic activity

Proteins were extracted from barley shoots and roots (48- to 120-h-old). Tissues were ground to a fine powder in liquid N_2_ using a mortar and pestle and then resuspended with five volumes of 50 mM KPi buffer (pH 7.5) to extract proteins. After centrifuging samples at 15,000×g for 15 min at 4°C, the supernatants were passed through a PD-10 column equilibrated with the same buffer. The eluate was used for enzyme assays. Protein concentrations were determined as described in the “Protein analysis” section. Enzyme reactions were performed as described in “Enzyme assay” section.

### *HvACT-2HS1* and *HvACT-2HL1/2* transcript analysis

Total RNA was purified from barley shoots and roots and reverse transcribed to cDNA as described in “Cloning of *HvACT-2HS1* cDNA” section. A quantitative PCR (qPCR) analysis was performed using 10 ng template cDNA, SYBR Premix Ex Taq (Takara Bio), and gene-specific primers (Supplementary Table S4). The PCR program, which was completed using a 7500 Real-Time PCR System (Applied Biosystems, Waltham, MA, USA), was as follows: 95°C for 30 s; 40 cycles of 95°C for 5 s and 60°C for 34 s. A standard curve was prepared using a dilution series of known quantities of plasmid containing *HvACT-2HS1* or *HvACT-2HL1* cDNA as the template. Amplification specificity was verified by a melting curve analysis and agarose gel electrophoresis.

## Results

### Identification of the 2HS chromosomal region responsible for *p*CA formation

**Figure 2:**
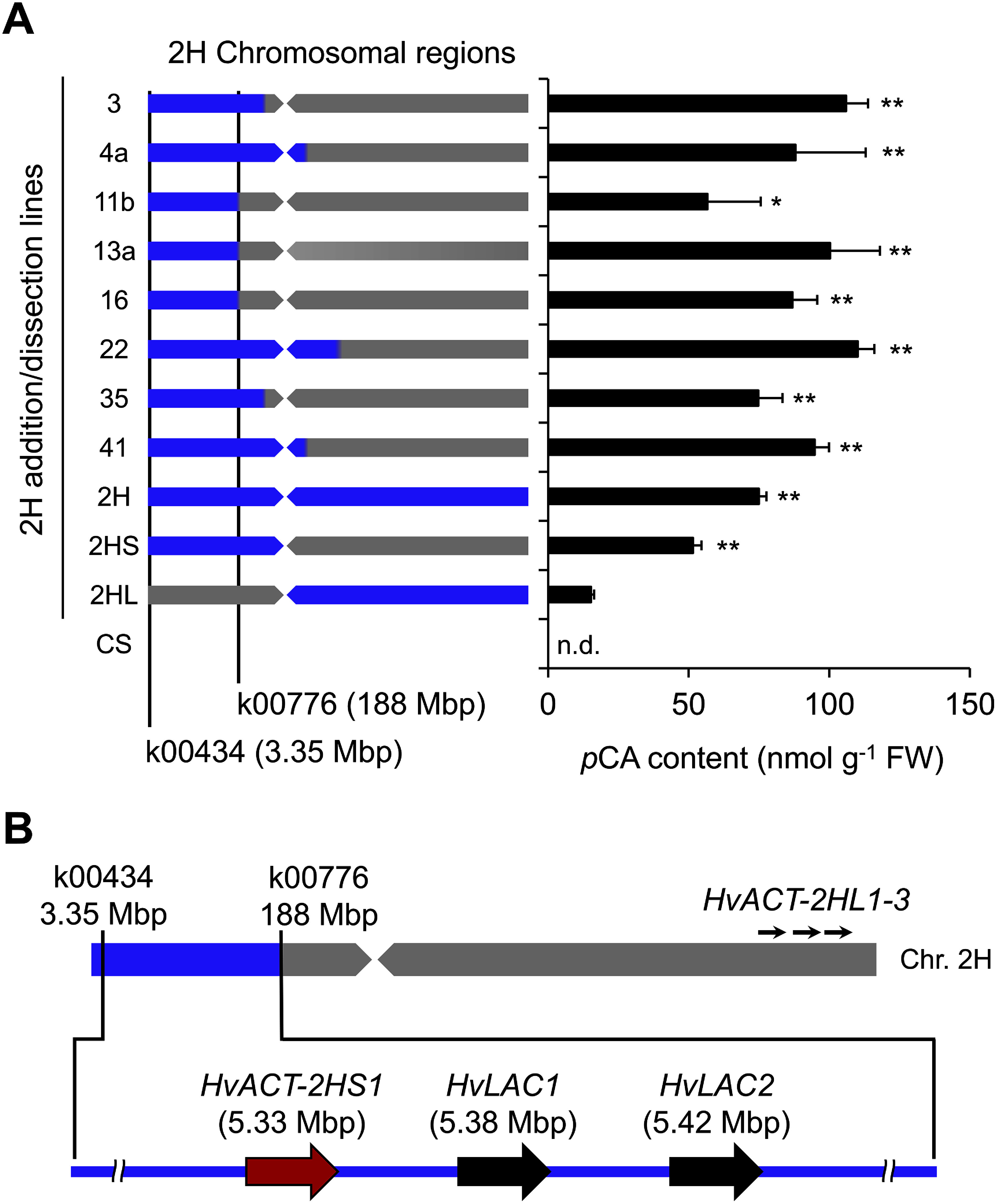
Figure 2. Occurrence of *p*-coumaroylagmatine (*p*CA) in wheat–barley 2H chromosome addition/dissection lines. (A) Chromosomal region added to the wheat (cv. ‘Chinese Spring’; CS) genetic background and the *p*CA content in 72-h-old shoots of each line. The 2H chromosomal region in each 2H addition/dissection line is presented in blue. Data are presented as the mean±SD (*n*=3). n.d.: not detected. The difference between 2HL addition line and the other 2H addition/dissection lines was tested using Dunnett’s test. * *p*<0.05; ** *p*<0.01 level. (B) Chromosomal location of EST markers and a candidate gene involved in *p*CA formation. The distal region of 2H short arm between EST markers (k00434 and k00776) is presented in blue. *HvACT-2HS1* is located in the distal region of the 2H short arm along with two laccase genes (*HvLAC1/2*). Three copies of the agmatine coumaroyltransferase gene (*HvACT-2HL1–3*) are located on the 2H long arm. See Supplementary Table S1 for EST markers.

An earlier chemical analysis of wheat–barley chromosome addition lines identified 2HS (in addition to 2HL) as the possible source of the gene responsible for *p*CA formation (Nomura et al. 1999, 2007). To narrow down the 2HS chromosomal region responsible for *p*CA formation, the *p*CA content in 72-h-old shoots was analyzed for dissection lines of wheat carrying aberrant 2H chromosomes as well as 2H, 2HS, and 2HL addition lines (Figure 2A). As described previously (Nomura et al. 1999, 2007), *p*CA accumulated in 2H, 2HS, and 2HL addition lines, but not in the parental wheat variety ‘Chinese Spring’ (CS). Additionally, *p*CA accumulated in all of the analyzed dissection lines (lines 3, 4a, 11b, 13a, 16, 22, 35, and 41). As described previously (Ube et al. 2023), HA accumulated in 2H and 2HS addition lines and these dissection lines, but not in 2HL addition line or CS (Supplementary Figure S2). The results indicated that the 2HS chromosomal region responsible for *p*CA formation is a 3.35–188 Mbp segment corresponding to the region between EST markers k00434 and k00776 (Figure 2B, Supplementary Table S1), which also includes *HvLAC1/2*. Therefore, we predicted that in addition to *HvACT-2HL* genes, this region contains another ACT-encoding gene.

### Selection of a candidate gene encoding ACT

A BLAST search of a barley sequence database (BARLEX; https://apex.ipk-gatersleben.de/apex/f?p=284:10) using the HvACT-2HL1 amino acid sequence as a query retrieved a putative ACT-encoding gene (*HvACT-2HS1*) located in the distal region of 2HS (3.35–188 Mbp) (Figure 2B). Interestingly, *HvACT-2HS1* is proximal to *HvLAC1*, which encodes a laccase that catalyzes the formation of HA from *p*CA. The HvACT-2HS1 amino acid sequence was similar to that of HvACT-2HL1 (46% sequence identity and 84% sequence similarity) (Supplementary Figure S3). The gene expression data in BARLEX indicated that *HvACT-2HS1* (HORVU2Hr1G002500.1) is highly expressed in 4-day-old embryos and developing grains (15 days after pollination) (Supplementary Table S2). This expression pattern is consistent with the accumulation of HA in barley seedlings and grains (Kohyama and Ono 2013; Ube et al. 2023), suggesting that *HvACT-2HS1* is likely involved in *p*CA formation. We cloned the full-length *HvACT-2HS1* cDNA sequence (GenBank accession no. LC877952) from 72-h-old shoots of barley cv. ‘Betzes’ and verified that it corresponds to the *HvACT-2HS1* genomic sequence in the barley genome database.

*HvACT-2HS1* encodes a polypeptide comprising 467 amino acids (Supplementary Figure S3), which is similar to the sequences of BAHD acyltransferase superfamily members, including known ACTs (D’Auria 2006; Petersen 2016; Yamane et al. 2021). The HvACT-2HS1 polypeptide possesses two conserved motifs (HXXXD and DFGWG) characteristic of this enzyme family. BAHD acyltransferases are phylogenetically sorted into several major clades. Some of the additional conserved motifs identified among BAHD acyltransferases are clade-specific motifs (Peng et al. 2016; Tuominen et al. 2011). Two Clade IV-specific motifs (VLWAFP and EVDSWL) were detected in the HvACT-2HS1 polypeptide. Furthermore, Clade IV has been divided into two subgroups (IVa and IVb) (Peng et al. 2016). The HvACT-2HS1 polypeptide contains two Clade IVa-specific motifs (VDGRAR and FMPSYLP), demonstrating that HvACT-2HS1 likely belongs to Clade IVa, similar to known ACTs.

**Figure 3:**
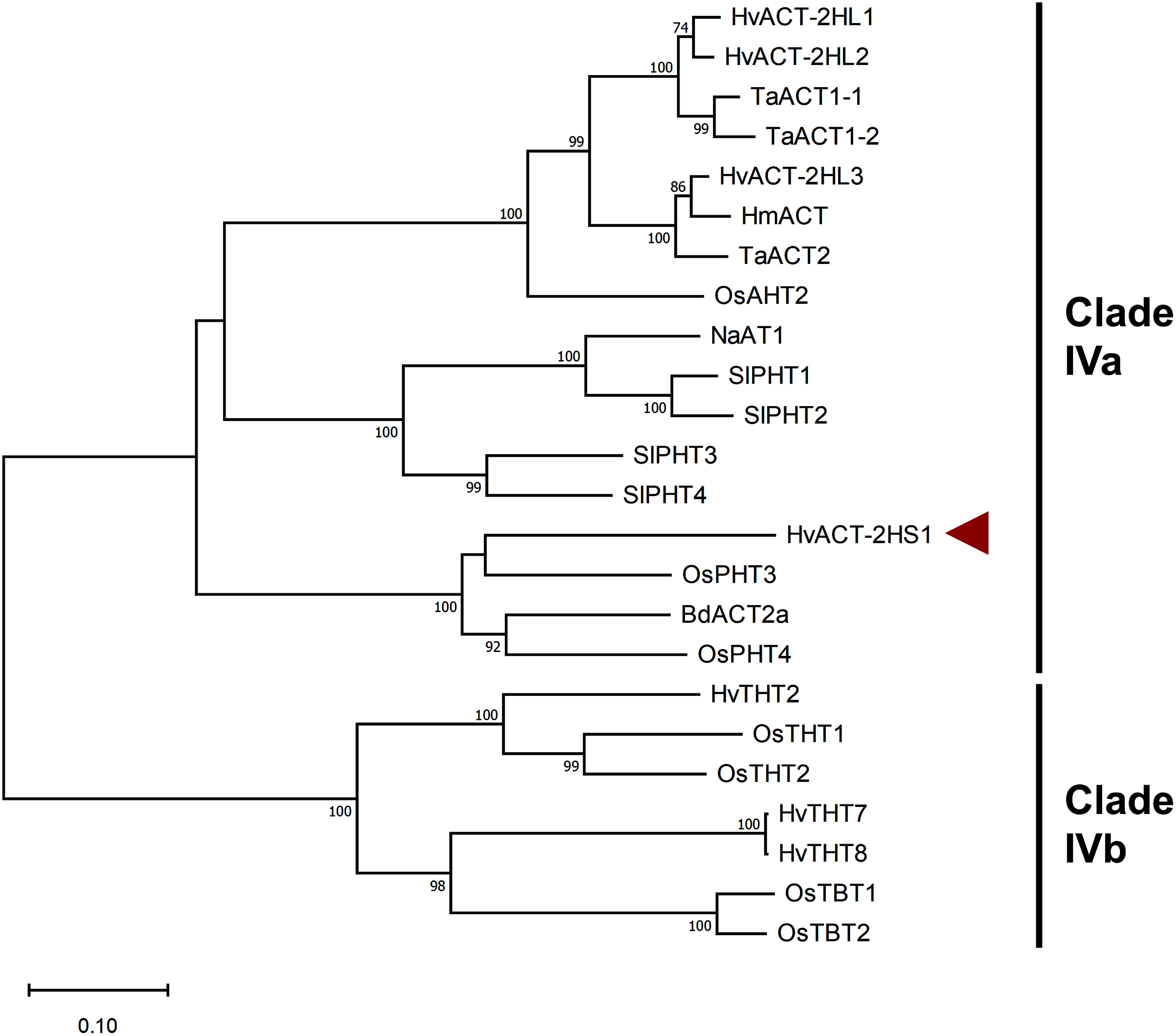
Figure 3. Phylogenetic relationships among HvACT-2HS1 and selected BAHD acyltransferase superfamily members in Clade IV. Amino acid sequences were aligned using ClustalW in MEGA, version 11 (Tamura et al. 2021). The unrooted dendrogram was constructed according to the neighbor-joining method with 1,000 bootstrap replicates using MEGA, version 11. Bootstrap values exceeding 70% are indicated at each node (bar=0.1 amino acid substitutions per site). HvACT-2HS1 identified in the present study is indicated by a red arrowhead. Taxa and gene codes of the sequences used for the analysis are listed in Supplementary Table S3.

A phylogenetic analysis of HvACT-2HS1 and sequences reportedly belonging to Clade IV enzymes (Figure 3, Supplementary Table S3) clearly showed that Clade IV includes ACT, agmatine hydroxycinnamoyltransferase (AHT), putrescine hydroxycinnamoyltransferase (PHT), tryptamine hydroxycinnamoyltransferase (THT), and tryptamine benzoyltransferase (TBT), which is in accordance with dendrogram of Clade IV constructed in a previous study (Yamane et al. 2021). HvACT-2HS1 was included in a group containing PHTs of rice (*Oryza sativa*) and ACT of *Brachypodium distachyon*, rather than in a group that included HvACT-2HL1/2. A comparison of amino acid sequences showed that HvACT-2HS1 is more similar to the closely related sequences OsPHT3, OsPHT4, and BdACT2a (69.5, 62.4, and 66.4% sequence identity, respectively) than to other ACTs/AHTs (44–48% sequence identity); OsPHT3/4 and BdACT2a catalyze the formation of hydroxycinnamic acid amides from agmatine and/or putrescine (Carere et al. 2018; Tanabe et al. 2016).

### Heterologous expression of recombinant HvACT-2HS1

To characterize its enzymatic function, HvACT-2HS1 was heterologously expressed in *E. coli*. SignalP (http://www.cbs.dtu.dk/services/SignalP/index.php (Accessed Nov 19, 2023)) predicted that the HvACT-2HS1 polypeptide lacks an N-terminal signal peptide like most BAHD acyltransferases. Thus, the full-length *HvACT-2HS1* sequence was used for the heterologous expression of a soluble recombinant HvACT-2HS1 with an N-terminal His-tag in *E. coli*. The recombinant protein was purified by metal-affinity chromatography. After removing the His-tag via thrombin digestion, His-tag-free HvACT-2HS1 was purified by metal-affinity chromatography and anion-exchange chromatography, with a yield of approximately 65 µg from 500 ml recombinant *E. coli* culture. The purified enzyme was detected as a 57-kDa band by SDS-PAGE analysis (Supplementary Figure S4). Its native molecular mass was estimated to be 51 kDa by gel-filtration, indicating that the recombinant enzyme exists as a monomer.

### Enzymatic characterization of recombinant HvACT-2HS1

**Figure 4:**
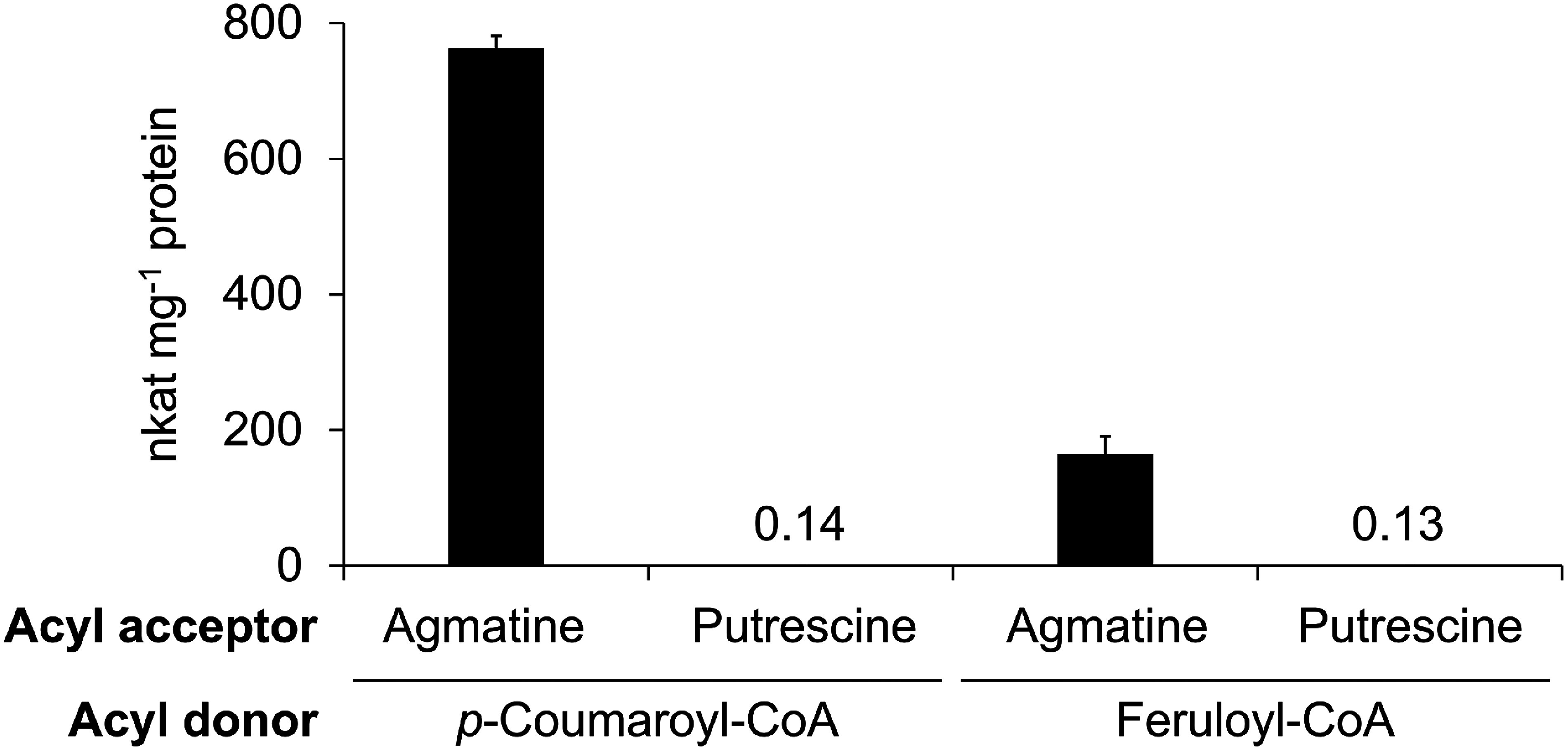
Figure 4. Specific activities of recombinant HvACT-2HS1. *p*-Coumaroyl-/feruloyl-CoAs and agmatine/putrescine were used as acyl donors and acceptors, respectively. Enzymatic activity was calculated on the basis of the formation of *p*CA, feruloylagmatine (FA), *p*-coumaroylputrescine (*p*CP), and feruloylputrescine (FP). Data are presented as the mean±SD (*n*=3).

We analyzed the enzymatic activity of the purified His-tag-free recombinant HvACT-2HS1 (i.e., condensation of *p*-coumaroyl-CoA and agmatine to form *p*CA) (Supplementary Figure S5A). HvACT-2HS1 catalyzed the formation of *p*CA, with a specific activity of 760 nkat mg^−1^ protein at saturating concentrations of substrates (100 µM *p*-coumaroyl-CoA and 100 µM agmatine) (Figure 4). The enzyme also catalyzed the formation of FA through the condensation of feruloyl-CoA and agmatine, with a specific activity of 170 nkat mg^−1^ protein at saturating concentrations of substrates (100 µM feruloyl-CoA and 100 µM agmatine) (Figure 4, Supplementary Figure S5B). However, only trace enzyme activities were detected for the formation of *p*-coumaroylputrescine (*p*CP) and feruloylputrescine (FP) (Figure 4, Supplementary Figure S5C, D). Thus, the enzyme preferentially accepts *p*-coumaroyl-/feruloyl-CoA as acyl donors and agmatine as the acyl acceptor.

The optimal pH and temperature of HvACT-2HS1 were 8.5 (Tris-HCl buffer) and 40°C, respectively (Supplementary Figure S6A, B). In addition, the enzyme was stable at a pH ranging from 5.5 to 10.0 (30 min at 25°C) and at temperatures of up to 45°C (30 min at pH 7.5) (Supplementary Figure S6C, D).

Apparent *K*_m_ and *k*_cat_ values of HvACT-2HS1 for acyl donors and acyl acceptors were determined when either the acyl donor or acceptor concentration was fixed (Table 1, Supplementary Figure S7). In the presence of 100 µM agmatine as the acyl acceptor, the *K*_m_ and *k*_cat_ values for *p*-coumaroyl-CoA (12 µM and 57 s^−1^, respectively) were approximately 2-fold and 4-fold higher, respectively, than those for feruloyl-CoA (6.6 µM and 15 s^−1^, respectively), resulting in an approximately 2-fold higher catalytic efficiency (*k*_cat_/*K*_m_) for *p*-coumaroyl-CoA than for feruloyl-CoA. In the presence of 100 µM *p*-coumaroyl-CoA as the acyl donor, the *K*_m_ and *k*_cat_ values for agmatine (30 µM and 67 s^−1^, respectively) were three orders of magnitude lower and approximately 40-fold higher, respectively, than those for putrescine (30,000 µM and 1.6 s^−1^, respectively), resulting in a 40,000-fold higher catalytic efficiency (*k*_cat_/*K*_m_) for agmatine than for putrescine. These results indicate that HvACT-2HS1 prefers *p*-coumaroyl-CoA over feruloyl-CoA as the acyl donor and agmatine over putrescine as the acyl acceptor. Moreover, the enzyme recognizes the structure of the acyl acceptor more strictly than that of the acyl donor.

**Table 1:** Table 1. Kinetic parameters of recombinant HvACT-2HS1.

Substrate	*K*_m_ (μM)	*k*_cat_ (s^−1^)	*k*_cat_/*K*_m_ (s^−1^ μM^−1^)
Acyl donors			
*p*-Coumaroyl-CoA^a^	12±1.3	57±2.3	4.7
Feruloyl-CoA^a^	6.6±2.2	15±1.4	2.3
Acyl acceptors			
Agmatine^b^	30±1.3	67±1.1	2.2
Putrescine^b^	30,000±2,200	1.6±0.0072	0.000055

Data are presented as the mean±SEM (SEM: error in data fitting to the Michaelis–Menten equation as calculated by a non-linear fitting program). Michaelis–Menten plots for each analysis are presented in Supplementary Figure S7. ^a^ 100 µM agmatine as the acyl acceptor. ^b^ 100 µM *p*-coumaroyl-CoA as the acyl donor. Values are different from those in Table 2. The data in this table are based on the enzyme reaction under optimal conditions for HvACT-2HS1. The data in Table 2 are based on the enzyme reaction under the same conditions as those used for HvACT-2HL1 (Nomura et al. 2018).

### Comparison of HvACT-2HS1 and HvACT-2HL1 enzymatic characteristics

To compare HvACT-2HS1 and HvACT-2HL1 in terms of their kinetic parameters, we determined the apparent *K*_m_ and *k*_cat_ values of the recombinant His-tag-free HvACT-2HS1 for acyl donors/acceptors under the same assay conditions as those used for the recombinant His-tag-free HvACT-2HL1 (Nomura et al. 2018) (Table 2, Supplementary Figure S8); the enzyme reactions were performed at 25°C in 100 mM Tris-HCl buffer (pH 7.5).

**Table 2:** Table 2. Kinetic parameters of recombinant HvACT-2HS1 and HvACT-2HL1.

Substrate	HvACT-2HS1			HvACT-2HL1^c^		
	*K*_m_ (μM)	*k*_cat_ (s^−1^)	*k*_cat_/*K*_m_ (s^−1^ μM^−1^)	*K*_m_ (μM)	*k*_cat_ (s^−1^)	*k*_cat_/*K*_m_ (s^−1^ μM^−1^)
Acyl donors						
*p*-Coumaroyl-CoA^a^	6.5±1.8	8.2±0.92	1.3	1.1±0.12	39±1.0	35
Feruloyl-CoA^a^	5.6±2.2	3.3±0.33	0.59	2.0±0.14	18±0.31	9.0
Acyl acceptors						
Agmatine^b^	24±2.1	8.7±0.34	0.36	1.7±0.13	38±0.87	22
Putrescine^b^	24,000±4,700	0.19±0.021	0.0000079	7,200±530	4.7±0.12	0.00065

Data are presented as the mean±SEM (SEM: error in data fitting to the Michaelis–Menten equation as calculated by a non-linear fitting program). Michaelis–Menten plots for each analysis are presented in Supplementary Figure S8. ^a^ 100 µM agmatine as the acyl acceptor. ^b^ 100 µM *p*-coumaroyl-CoA as the acyl donor. ^c^ Data are from Nomura et al. (2018). Values for HvACT-2HS1 are different from those in Table 1. The data in this table are based on the enzyme reaction under the same conditions as those used for HvACT-2HL1 (Nomura et al. 2018). The data in Table 1 are based on the enzyme reaction under optimal conditions for HvACT-2HS1.

In the presence of 100 µM agmatine as the acyl acceptor, the *K*_m_ and *k*_cat_ values of HvACT-2HS1 for acyl donors (*p*-coumaroyl-CoA, 6.5 µM and 8.2 s^−1^, respectively; feruloyl-CoA, 5.6 µM and 3.3 s^−1^, respectively) were higher and lower, respectively, than those of HvACT-2HL1 (*p*-coumaroyl-CoA, 1.1 µM and 39 s^−1^, respectively; feruloyl-CoA, 2.0 µM and 18 s^−1^, respectively). Thus, compared with HvACT-2HL1, HvACT-2HS1 had lower catalytic efficiencies (*k*_cat_/*K*_m_) for *p*-coumaroyl-CoA (1/27) and feruloyl-CoA (1/15). In the presence of 100 µM *p*-coumaroyl-CoA as the acyl donor, the *K*_m_ and *k*_cat_ values of HvACT-2HS1 for acyl acceptors (agmatine, 24 µM and 8.7 s^−1^, respectively; putrescine, 24,000 µM and 0.19 s^−1^, respectively) were higher and lower, respectively, than those of HvACT-2HL1 (agmatine, 1.7 µM and 38 s^−1^, respectively; putrescine, 7,200 µM and 4.7 s^−1^, respectively). Therefore, compared with HvACT-2HL1, HvACT-2HS1 had lower catalytic efficiencies (*k*_cat_/*K*_m_) for agmatine (1/61) and putrescine (1/82). These results suggest that both HvACT-2HS1 and HvACT-2HL1 prefer *p*-coumaroyl-CoA and agmatine as the acyl donor and acyl acceptor, respectively, and HvACT-2HS1 has lower *p*CA-forming activity than HvACT-2HL1.

### Profiles of *HvACT-2HS1* and *HvACT-2HL1/2* transcription, *p*CA-forming enzymatic activity, and *p*CA accumulation in barley seedlings

**Figure 5:**
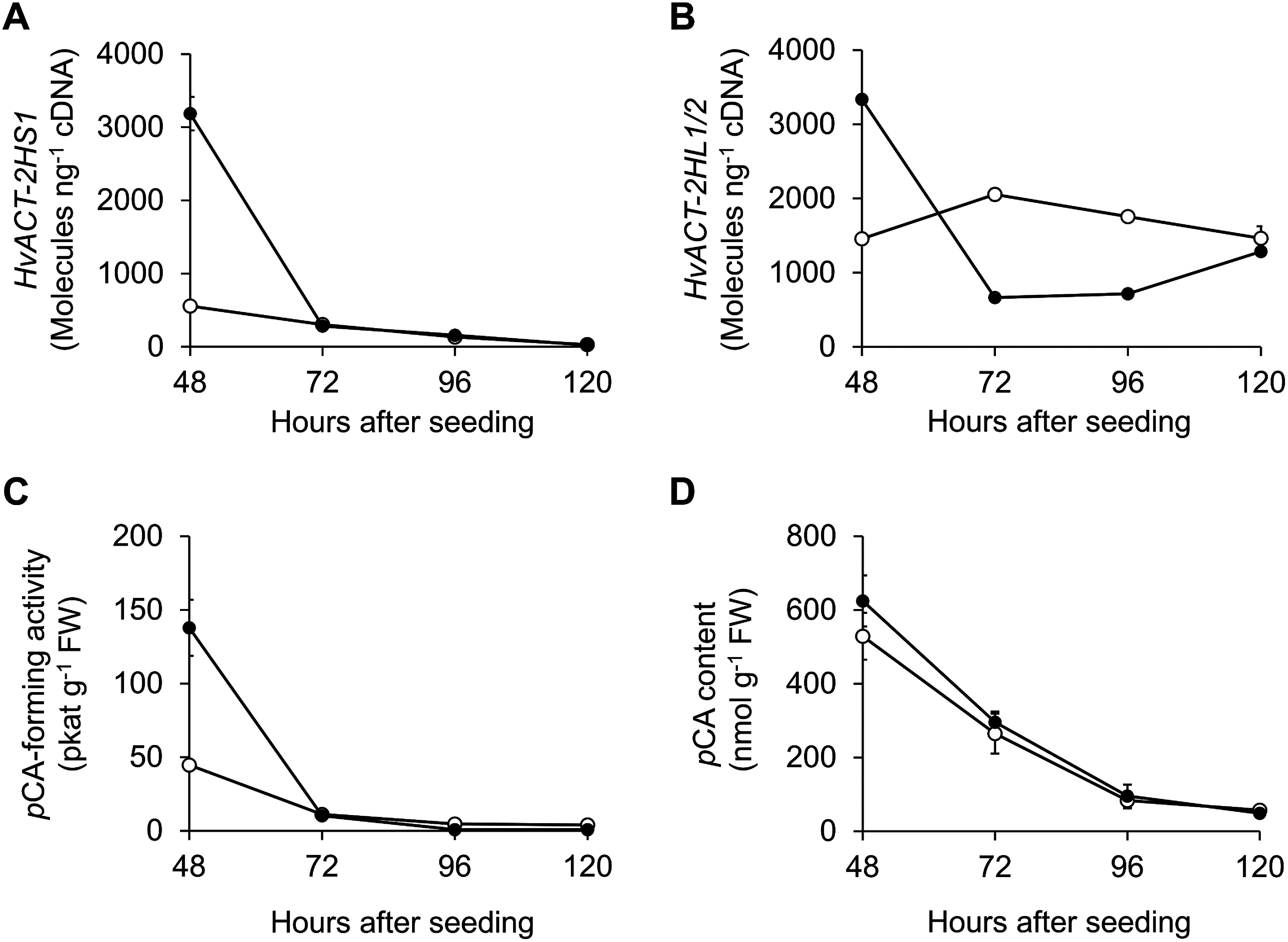
Figure 5. Time-course changes in *HvACT-2HS1* and *HvACT-2HL1/2* transcript levels, *p*CA-forming enzymatic activity, and *p*CA content in barley seedlings. (A) *HvACT-2HS1* transcript levels. (B) *HvACT-2HL1/2* transcript levels. (C) *p*CA-forming activity in the crude enzyme extract. (D) *p*CA content. The shoots and roots of barley (48- to 120-h-old; Supplementary Figure S1) were used for these analyses. Filled circles, shoots; empty circles, roots. Data are presented as the mean±SD (*n*=3).

Time-course changes in *HvACT-2HS1* and *HvACT-2HL1/2* transcript levels in barley seedlings were examined by RT-qPCR analysis. Because *HvACT-2HL1* and *HvACT-2HL2* have almost identical nucleotide sequences, their transcript levels were measured collectively using a single primer set. In shoots, *HvACT-2HS1* and *HvACT-2HL1/2* transcript levels varied almost in parallel; levels were highest soon after germination (48 h), after which they decreased in a time-dependent manner (Figure 5A, B). *HvACT-2HS1* transcript levels were lower in roots than in shoots throughout the experimental period. Additionally, *HvACT-2HL1/2* transcript levels were higher than those of *HvACT-2HS1* and were almost unchanged throughout the experimental period. Changes in the *p*CA-forming enzymatic activity (Figure 5C) and *p*CA content (Figure 5D) in shoots and roots were almost in accordance with the *HvACT-2HS1* and *HvACT-2HL1/2* transcript profiles. At 48 h, shoots and roots had similar *p*CA contents, whereas *HvACT-2HS1* and *HvACT-2HL1/2* transcript levels were higher in shoots than in roots. Recent research showed that HA is more abundant in shoots than in roots (Ube et al. 2023). Accordingly, *p*CA formed by HvACTs is likely converted to HA more actively in shoots than in roots.

## Discussion

According to previous studies, HvACT-2HL1/2, which are encoded by genes on 2HL, are involved in the formation of *p*CA for HA biosynthesis in barley seedlings (Nomura et al. 2007, 2018). However, on the basis of an analysis of *p*CA accumulation in wheat–barley chromosome addition lines, it was predicted that another ACT-encoding gene is on 2HS (Nomura et al. 2007). In the present study involving a chemical analysis of cytogenetically generated barley chromosome dissection lines of wheat, we narrowed down the chromosomal region responsible for *p*CA formation to the distal region of 2HS, while also identifying an ACT-encoding gene (*HvACT-2HS1*) proximal to *HvLAC1*. The characterization of recombinant HvACT-2HS1 demonstrated that the enzyme catalyzes *p*CA formation with high specificity for agmatine as the acyl acceptor. According to the study findings, in addition to HvACT-2HL1/2, the isozyme HvACT-2HS1 contributes to the formation of *p*CA for HA biosynthesis in barley.

ACTs have been identified not only from barley but also from other gramineous plants, including *Hordeum murinum*, wheat and rice (Peng et al. 2016; Tanabe et al. 2016; Yamane et al. 2021). Yamane et al. (2021) noted that ACTs can be divided into two groups on the basis of the substrate specificities; group 1 enzymes show comparable catalytic efficiency for *p*-coumaroyl- and feruloyl-CoAs, while group 2 enzymes prefer feruloyl-CoA over *p*-coumaroyl-CoA. Moreover, it was indicated that group 1 enzymes exhibit significantly higher affinity for agmatine than group 2 enzymes. According to these criteria, HvACT-2HS1, which shows approximately 2-fold higher catalytic efficiency for *p*-coumaroyl-CoA than for feruloyl-CoA and approximately 40,000-fold higher catalytic efficiency for agmatine than for putrescine, belongs to group 1. This is also applicable to HvACT-2HL1 (Nomura et al. 2018).

The *K*_m_ value of HvACT-2HS1 for *p*-coumaroyl-CoA (12 µM) was approximately 10-fold higher than those of reported ACTs in Triticeae (i.e., barley, wheat, and *H. murinum*), which are approximately 1 µM (Nomura et al. 2018; Yamane et al. 2021). This relatively low affinity of HvACT-2HS1 toward *p*-coumaroyl-CoA appears to be compensated by the high *k*_cat_ value (57 s^−1^), resulting in the catalytic efficiency (4.7 s^−1^ μM^−1^) comparable to those of other ACTs except for HvACT-2HL1, having remarkably high catalytic efficiency (35 s^−1^ μM^−1^; Nomura et al. 2018).

Analyses of *HvACT-2HS1* and *HvACT-2HL1/2* transcript profiles in barley seedlings demonstrated that these genes were similarly transcribed in shoots. However, in roots, *HvACT-2HS1* transcript levels were lower than those of *HvACT-2HL1/2*. These results suggest that *p*CA formation is mediated by HvACT-2HS1 and HvACT-2HL1/2 in shoots, but by only HvACT-2HL1/2 in roots. Considering that HvACT-2HL1 had a significantly higher catalytic efficiency for *p*CA formation than HvACT-2HS1 (Table 2), it is likely that HvACT-2HL1/2 mainly contribute to *p*CA formation, with HvACT-2HS1 playing a supporting role. On the other hand, *p*CA content in 2H addition/dissection wheat lines carrying the distal region of 2HS was higher than that in 2HL addition line, which implies that HvACT-2HS1 rather than HvACT-2HL1/2 plays the major role in barley. A possible explanation for this discrepancy is that the expression levels of barley genes in the addition/dissection lines differ from those in barley, because the addition/dissection lines inherently have genetic background of wheat. To reveal how much extent HvACT-2HS1 and HvACT-2HL1/2 contribute to *p*CA formation, the quantitative analysis of contents of *p*CA, as well as HA, using knockout lines of each *ACT* gene will be needed.

The *HvACT-2HS1* transcript profile was more similar to those of *HvLAC1/2* than to those of *HvACT-2HL1/2*; *HvLAC1/2* are highly transcribed in shoots, but are transcribed at low levels in roots (Ube et al. 2023), suggesting that *HvACT-2HS1* and *HvLAC1/2* are co-expressed genes. Many biosynthetic genes of specific secondary metabolites have been reported; these genes are located in specific genomic regions ranging from ten to several hundred kilobase pairs (Nützmann and Osbourn 2014). These co-localized biosynthetic genes are usually co-expressed by the same regulatory mechanism, leading to the sequential formation of specific metabolites. Thus, the presence of a metabolic gene cluster comprising *HvACT-2HS1* and *HvLAC1/2* in a specific region (100 kbp) of 2HS (Figure 2B) may be conducive to efficient HA formation in barley.

However, the inclusion of *HvACT-2HS1* rather than *HvACT-2HL1/2* in the gene cluster with *HvLAC1/2* may not be ideal for HA production because the *p*CA-forming enzymatic activity of HvACT-2HS1 is much lower than that of HvACT-2HL1/2. According to a phylogenetic analysis, HvACT-2HS1 is closely related to rice PHTs (OsPHT3/4). *OsPHT3/4* are located on chromosome 9A of rice. Considering the lack of synteny between rice chromosome 9A and barley chromosome 2H, *HvACT-2HS1* does not have an orthologous relationship with *OsPHT3/4*. A search of the barley genome database using HvACT-2HS1 as a query revealed four sequences homologous to HvACT-2HS1 (67.5–71.0% amino acid sequence identity); genes encoding these homologous sequences were detected on barley chromosome 5H (Supplementary Table S5). Because the chromosomal position of three *HvACT-2HS1* homologs corresponded to the syntenic region (i.e., 346–517 Mbp on 5H) with rice chromosome 9A (Thiel et al. 2009), *HvACT-2HS1* homologs on chromosome 5H appear to be orthologs of *OsPHT3/4.* On the basis of the similarity between HvACT-2HS1 and HvACT-2HL1 (only 46% sequence identity), *HvACT* genes on 2HL are not the paralogs of *HvACT-2HS1*. Instead, they originated from a different ancestral gene. Indeed, HvACT-2HL1/2 are more closely related to rice AHT (OsAHT2) than to HvACT-2HS1 or OsPHT3/4 (Figure 3). *OsAHT2* is located on rice chromosome 4A, which has a syntenic relationship with barley chromosome 2H (Thiel et al. 2009), suggesting that *HvACT-2HL1/2* are orthologs of *OsAHT2*. Thus, we propose the following evolutionary scenario: the ancestors of *OsPHT3/4* orthologs and *OsAHT2* orthologs had individually been present in a common ancestor of rice and barley, because the orthologs are commonly distributed in rice, barley, and other poaceous plants (Figure 3). The former had the property to accept both agmatine and putrescine as acyl acceptor, and the latter had the property to accept agmatine, on the basis of the reported substrate specificities of OsPHT3 (i.e., putrescine type), OsPHT4 (i.e., agmatine and putrescine type) and OsAHT2 (i.e., agmatine type). Then, the substrate specificity of *OsPHT3* orthologs had gradually been refined for agmatine or putrescine during evolution of these poaceous species, while the substrate specificity of *OsPHT4* orthologs had been retained. In particular, during the evolution of barley, one of the *OsPHT3* orthologs on chromosome 5H was duplicated and recruited into the metabolic gene cluster on 2HS. Therefore, it is most likely that *HvACT-2HS1* and *HvACT-2HL1/2*, which originated from different ancestral genes, jointly mediate the formation of *p*CA for HA biosynthesis in barley.

## Abbreviations

2HL: long arm of barley chromosome 2H
2HS: short arm of barley chromosome 2H
ACT: agmatine coumaroyltransferase
AHT: agmatine hydroxycinnamoyltransferase
Bd: *Brachypodium distachyon*
FA: feruloylagmatine
FP: feruloylputrescine
FW: fresh weight
HA: hordatine A
HB: hordatine B
HC: hordatine C
Hv: *Hordeum vulgare*
LAC: laccase
ODS: octadecylsilyl
Os: *Oryza sativa*
*p*CA: *p*-coumaroylagmatine
*p*CP: *p*-coumaroylputrescine
PHT: putrescine hydroxycinnamoyltransferase
TBT: tryptamine benzoyltransferase
THT: tryptamine hydroxycinnamoyltransferase

## Accession numbers

The nucleotide sequence reported in this article has been submitted to GenBank/EMBL/DDBJ databases, with the following accession number: LC877952 (*HvACT-2HS1* cDNA).

## Description of supplementary files

Supplementary Table S1. Physical positions of EST markers in wheat–barley 2H chromosome dissection lines.

Supplementary Table S2. Details regarding a candidate gene for *ACT* on 2HS (*HvACT-2HS1*).

Supplementary Table S3. Genes used for a phylogenetic analysis.

Supplementary Table S4. Primer sequences used in this study.

Supplementary Table S5. Details regarding *HvACT-2HS1* homologs in the barley genome.

Supplementary Figure S1. Appearance of barley seedlings after seeding.

Supplementary Figure S2. Occurrence of hordatine A (HA) in wheat–barley 2H chromosome addition/dissection lines.

Supplementary Figure S3. Alignment of HvACT-2HS1 and HvACT-2HL1 amino acid sequences.

Supplementary Figure S4. Purification of recombinant HvACT-2HS1 expressed in *Escherichia coli*.

Supplementary Figure S5. Enzymatic activities of recombinant HvACT-2HS1 for the formation of *p*-coumaroylagmatine (*p*CA), feruloylagmatine (FA), *p*-coumaroylputrescine (*p*CP), and feruloylputrescine (FP).

Supplementary Figure S6. Effects of pH and temperature on enzyme activity and stability of recombinant HvACT-2HS1.

Supplementary Figure S7. Michaelis–Menten plots for reactions catalyzed by recombinant HvACT-2HS1 under optimal conditions.

Supplementary Figure S8. Michaelis–Menten plots for reactions catalyzed by recombinant HvACT-2HS1 under the same conditions as those used for HvACT-2HL1.
