# Supplementary material for: Agmatine coumaroyltransferase gene on the short arm of chromosome 2H is involved in hordatine biosynthesis in barley
Source: Plant Biotechnol (Tokyo). 2026 Mar 25;43(1):105–15. doi: 10.5511/plantbiotechnology.25.1209a (PMC13170813; doi:10.5511/plantbiotechnology.25.1209a)
Supplement: Supplementary Data [file plantbiotechnology-43-1-25.1209a-s001.pdf]

Supplementary Information

For:

Agmatine coumaroyltransferase gene on the short arm of chromosome 2H is involved in hordatine biosynthesis in barley

Naoki Ube[1,*], Taiji Nomura[1]

[1]Biotechnology Research Center and Department of Biotechnology, Toyama Prefectural University, 5180 Kurokawa, Imizu, Toyama 939-0398, Japan

*Corresponding author. Tel.: +81-766-56-7500 (ext. 1517)

E-mail address: nube@pu-toyama.ac.jp

Supplementary Table S1. Physical positions of EST markers in wheat–barley 2H chromosome dissection lines

	EST marker	Position on 2H[a]	Line number							
			3	4a	11b	13a	16	22	35	41
2HS	k00434	3346664	+	+	+	+	+	+	+	+
	k01312	3989249	+	+	+	+	+	+	+	+
	k04935	5116018	+	+	+	+	+	+	+	+
	k04102	8528758	+	+	+	+	+	+	+	+
	k00074	11394521	+	+	+	+	+	+	+	+
	k04909	13265819	+	+	+	+	+	+	+	+
	k01216	17231509	+	+	+	+	+	+	+	+
	k01603	21580812	+	+	+	+	+	+	+	+
	k04629	27173722	+	+	+	+	+	+	+	+
	k00144	27992852	+	+	+	+	+	+	+	+
	k04267	32711201	+	+	+	+	+	+	+	+
	k01253	33897960	+	+	+	+	+	+	+	+
	k04773	51429837	+	+	+	+	+	+	+	+
	k05056	53907569	+	+	+	+	+	+	+	+
	k02521	57285501	+	+	+	+	+	+	+	+
	k04763	64607629	+	+	+	+	+	+	+	+
	k03601	68833703	+	+	+	+	+	+	+	+
	k03341	74786755	+	+	+	+	+	+	+	+
	k04377	78366494	+	+	+	+	+	+	+	+
	k03538	92522775	+	+	+	+	+	+	+	+
	k00313	110653968	+	+	+	+	+	+	+	+
	k03436	113455676	+	+	+	+	+	+	+	+
	k03467[b]	-	+	+	+	+	+	+	+	+
	k00757	140216911	+	+	+	+	+	+	+	+
	k00852	146746516	+	+	+	+	+	+	+	+
	k00186	161710895	+	+	+	+	+	+	+	+
	k00265	168268200	+	+	+	+	+	+	+	+
	k01238	185336652	+	+	+	+	+	+	+	+
	k05033	185337465	+	+	+	+	+	+	+	+
	k00776	188388474	+	+	+	+	+	+	+	+
	k00376	191451486	+	+	−	−	−	+	+	+
	k00168	192200352	+	+	−	−	−	+	+	+
	k01932	198408879	+	+	−	−	−	+	+	+
	k01457	208816709	+	+	−	−	−	+	+	+
	k03332	214952675	+	+	−	−	−	+	+	+
	k01360	219420724	+	+	−	−	−	+	+	+
	k03744	232635768	+	+	−	−	−	+	+	+
	k04039	235587746	+	+	−	−	−	+	+	+
	k04759	237277381	+	+	−	−	−	+	+	+
	k03300	239777839	+	+	−	−	−	+	+	+
	k00677	248455513	+	+	−	−	−	+	+	+
	k02630	248455515	+	+	−	−	−	+	+	+
	k00091	252052778	+	+	−	−	−	+	+	+
	k00838	255503723	+	+	−	−	−	+	+	+
	k02202	267303703	+	+	−	−	−	+	+	+
	k00363	287332666	+	+	−	−	−	+	+	+
	k02121	318874372	+	+	−	−	−	+	+	+
	k04415	322916922	−	+	−	−	−	+	−	+

Supplementary Table S1. (*continued*)

2HL	k03501	361265831	−	+	−	−	−	+	−	+
	k01407	366460123	−	+	−	−	−	+	−	+
	k03201	379412457	−	+	−	−	−	+	−	+
	k04365	417215615	−	+	−	−	−	+	−	+
	k03186	466479861	−	+	−	−	−	+	−	+
	k04142	477169224	−	+	−	−	−	+	−	+
	k04286	478879129	−	−	−	−	−	+	−	−
	k03195	503886922	−	−	−	−	−	+	−	−
	k00679	525250388	−	−	−	−	−	+	−	−
	k00132	534370445	−	−	−	−	−	+	−	−
	k03404	535279939	−	−	−	−	−	+	−	−
	k00491	539003428	−	−	−	−	−	+	−	−
	k03409	541870571	−	−	−	−	−	+	−	−
	k02551	545216751	−	−	−	−	−	+	−	−
	k03231	570135038	−	−	−	−	−	+	−	−
	k00246	588115586	−	−	−	−	−	+	−	−
	k03187	588247961	−	−	−	−	−	+	−	−
	k01321	592677641	−	−	−	−	−	+	−	−
	k01579	621114608	−	−	−	−	−	+	−	−
	k04988	621211485	−	−	−	−	−	−	−	−
	k01073	633255648	−	−	−	−	−	−	−	−
	k00908	636434765	−	−	−	−	−	−	−	−
	k04003	640232936	−	−	−	−	−	−	−	−
	k03443	640776038	−	−	−	−	−	−	−	−
	k04939	645202738	−	−	−	−	−	−	−	−
	k03160	645284915	−	−	−	−	−	−	−	−
	k05173	646939653	−	−	−	−	−	−	−	−
	k02590	647547013	−	−	−	−	−	−	−	−
	k02245	648513722	−	−	−	−	−	−	−	−
	k01019	649447445	−	−	−	−	−	−	−	−
	k00289	651374673	−	−	−	−	−	−	−	−
	k01362	659264429	−	−	−	−	−	−	−	−
	k01219	663533204	−	−	−	−	−	−	−	−
	k01086	664191820	−	−	−	−	−	−	−	−
	k04929	677483121	−	−	−	−	−	−	−	−
	k00998	685490300	−	−	−	−	−	−	−	−
	k00748	687579443	−	−	−	−	−	−	−	−
	k01975	687999309	−	−	−	−	−	−	−	−
	k02482	688034188	−	−	−	−	−	−	−	−
	k04782	688520322	−	−	−	−	−	−	−	−
	k04784	688527548	−	−	−	−	−	−	−	−
	k04771	690494723	−	−	−	−	−	−	−	−
	k00777	691396406	−	−	−	−	−	−	−	−
	k03622	694293709	−	−	−	−	−	−	−	−
	k03454	696112060	−	−	−	−	−	−	−	−
	k00314	700396801	−	−	−	−	−	−	−	−
	k01170	702542113	−	−	−	−	−	−	−	−
	k03376	710602937	−	−	−	−	−	−	−	−
	k02580	712310244	−	−	−	−	−	−	−	−
	k05037	720345054	−	−	−	−	−	−	−	−
	k04107	721300417	−	−	−	−	−	−	−	−
	k03899	722484163	−	−	−	−	−	−	−	−
	k04446	725822856	−	−	−	−	−	−	−	−
	k01224	732791728	−	−	−	−	−	−	−	−
	k01408	735300342	−	−	−	−	−	−	−	−
	k01418	735881660	−	−	−	−	−	−	−	−
	k03044	738323621	−	−	−	−	−	−	−	−
	k01467	740673980	−	−	−	−	−	−	−	−
	k04721	744765163	−	−	−	−	−	−	−	−
	k03085	747405286	−	−	−	−	−	−	−	−
	k01423	747945144	−	−	−	−	−	−	−	−
	k00323	749602678	−	−	−	−	−	−	−	−
	k00730	752356830	−	−	−	−	−	−	−	−
	k03370	758621062	−	−	−	−	−	−	−	−
	k00579	759163032	−	−	−	−	−	−	−	−
	k00932	761249809	−	−	−	−	−	−	−	−
	k03626	767055591	−	−	−	−	−	−	−	−

"+" and "−" indicate presence and absence of PCR amplicon, respectively, during a genomic PCR analysis using EST markers (Joshi et al. 2011).
[a]Position of EST markers as determined by a BLAST search of the BARLEX database using forward primer sequences as described by Joshi et al. (2011).
[b]k03467 is located near k00313 and k00852 (Joshi et al. 2011), but the database search indicated it was on an unknown chromosome.

Supplementary Table S2. Details regarding a candidate gene for *ACT* on 2HS (*HvACT-2HS1*)

	HvACT-2HS1
Gene code in BARLEX	HORVU2Hr1G002500.1
Chromosome	2H
Position	5332106–5333847
Annotation	HXXXD-type acyl-transferase family protein
Tissue/growth stage	FPKM[a]
EMB[b]	96
ROO1	2.3
LEA	0.71
INF1	0
INF2	0.51
NOD	0.012
CAR5	0.76
CAR15	13
ETI	8.0
LEM	16
LOD	31
PAL	23
EPI	1.6
RAC	33
ROO2	25
SEN	0.025

[a]FPKM (fragments per kilobase of exon per million reads mapped) values were obtained from the BARLEX database.
[b]Abbreviations indicate barley tissues and growth stages used for analyzing gene expression:
EMB: embryos (4 days after seeding)
ROO1: roots from seedlings (10 cm shoot stage)
LEA: shoots from seedlings (10 cm shoot stage)
INF1: young developing inflorescences (5 mm)
INF2: developing inflorescences (1–1.5 cm)
NOD: developing tillers, 3rd internode (42 days after pollination, DAP)
CAR5: developing grains (5 DAP)
CAR15: developing grains (15 DAP)
ETI: etiolated seedlings, dark condition (10 days after seeding)
LEM: inflorescences, lemma (42 DAP)
LOD: inflorescences, lodicule (42 DAP)
PAL: dissected inflorescences, palea (42 DAP)
EPI: epidermal strips (28 DAP)
RAC: inflorescences, rachis (35 DAP)
ROO2: roots (28-day-old seedlings)
SEN: senescing leaves (56 DAP)

Supplementary Table S3. Genes used for a phylogenetic analysis

Name	Taxon	Gene code	Reference
HvACT-2HS1	*Hordeum vulgare*	LC877952	This study
HvACT-2HL1	*Hordeum vulgare*	AB334132	Nomura et al. (2007)
HvACT-2HL2	*Hordeum vulgare*	AB334133	Nomura et al. (2007)
HvACT-2HL3	*Hordeum vulgare*	BAK00935	Yamane et al. (2021)
HvTHT2	*Hordeum vulgare*	HORVU1Hr1G019410	Ube et al. (2019)
HvTHT7	*Hordeum vulgare*	HORVU4Hr1G077780	Ube et al. (2019)
HvTHT8	*Hordeum vulgare*	HORVU4Hr1G077790	Ube et al. (2019)
HmACT	*Hordeum murinum*	LC596950	Yamane et al. (2021)
TaACT1-1	*Triticum aestivum*	LC596951	Yamane et al. (2021)
TaACT1-2	*Triticum aestivum*	LC596952	Yamane et al. (2021)
TaACT2	*Triticum aestivum*	KT962210	Yamane et al. (2021)
BdACT2a	*Brachypodium distachyon*	XM_003578512	Carere et al. (2018)
OsAHT2	*Oryza sativa*	Os04g0664600	Tanabe et al. (2016)
OsPHT3	*Oryza sativa*	Os09g0543900	Tanabe et al. (2016)
OsPHT4	*Oryza sativa*	Os09g0544000	Tanabe et al. (2016)
OsTBT1	*Oryza sativa*	KX430021	Peng et al. (2016)
OsTBT2	*Oryza sativa*	KX430022	Peng et al. (2016)
OsTHT1	*Oryza sativa*	KX430019	Peng et al. (2016)
OsTHT2	*Oryza sativa*	KX430020	Peng et al. (2016)
NaAT1	*Nicotiana attenuata*	JN390826	Onkokesung et al. (2012)
SlPHT1	*Solanum lycopersicum*	ON248950	Roumani et al. (2025)
SlPHT2	*Solanum lycopersicum*	ON248951	Roumani et al. (2025)
SlPHT3	*Solanum lycopersicum*	OP296537	Roumani et al. (2025)
SlPHT4	*Solanum lycopersicum*	OP296536	Roumani et al. (2025)

Supplementary Table S4. Primer sequences used in this study

Primer name	Sequence (5′ to 3′)
For cloning *HvACT-2HS1*	
HvACT-2HS1/pET28a-F	CGCGCGGCAGCCATATGGAGGTCGCTAGCTCACC
HvACT-2HS1/pET28a-R	GTCATGCTAGCCATATCATGCATGGAGATCATCG
For RT-qPCR	
HvACT-2HS1 rt-F	GACGCCATCGTACATCATGC
HvACT-2HS1 rt-R	CATTGCTTGAAAGCCTCGAG
HvACT-2HL1/2 rt-F	CTTCTTCATGCCCAGCTACC
HvACT-2HL1/2 rt-R	CTTGAAGGTGTTCATGTCGC

"F" and "R" indicate "forward" and "reverse", respectively.

Supplementary Table S5. Details regarding *HvACT-2HS1* homologs in the barley genome

	HORVU5Hr1G077720.1	HORVU5Hr1G079580.1	HORVU5Hr1G084810.1	HORVU5Hr1G084820.2
Chromosome	5H	5H	5H	5H
Position	493321559–493323277	501592389–501593699	573348016–573349459	506846707–506848370
Annotation	HXXXD-type acyl-transferase family protein	HXXXD-type acyl-transferase family protein	HXXXD-type acyl-transferase family protein	HXXXD-type acyl-transferase family protein
Amino acid identity/similarity with HvACT-2HS1	67.5%/92.1%	67.5%/92.1%	71.0%/92.4%	70.0%/93.1%
Tissue/growth stage	FPKM[a]			
EMB[b]	0	0	0	1.1
ROO1	0	0	0.19	8.0
LEA	0	0	0	0.3
INF1	0	0	0	0
INF2	0	0	0	0
NOD	0	0	0.072	9.0
CAR5	0	0	0	0.024
CAR15	0	0	0	0.073
ETI	0	0.29	0.061	3.6
LEM	0	0	0.10	0.40
LOD	0	0	0.056	0.19
PAL	0	0.0086	0.19	0.88
EPI	0	0	0	0.18
RAC	0	0	0.056	3.6
ROO2	0	0.19	2.6	7.9
SEN	0	0	0.010	0.018

Listed genes encode proteins similar to HvACT-2HS1 (>60% amino acid sequence identity) according to a BLAST search of the BARLEX database.
[a]FPKM (fragments per kilobase of exon per million reads mapped) values were obtained from the BARLEX database.
[b]Abbreviations indicate barley tissues and growth stages used for analyzing gene expression:
EMB: embryos (4 days after seeding)
ROO1: roots from seedlings (10 cm shoot stage)
LEA: shoots from seedlings (10 cm shoot stage)
INF1: young developing inflorescences (5 mm)
INF2: developing inflorescences (1–1.5 cm)
NOD: developing tillers, 3rd internode (42 days after pollination, DAP)
CAR5: developing grains (5 DAP)
CAR15: developing grains (15 DAP)
ETI: etiolated seedlings, dark condition (10 days after seeding)
LEM: inflorescences, lemma (42 DAP)
LOD: inflorescences, lodicule (42 DAP)
PAL: dissected inflorescences, palea (42 DAP)
EPI: epidermal strips (28 DAP)
RAC: inflorescences, rachis (35 DAP)
ROO2: roots (28-day-old seedlings)
SEN: senescing leaves (56 DAP)

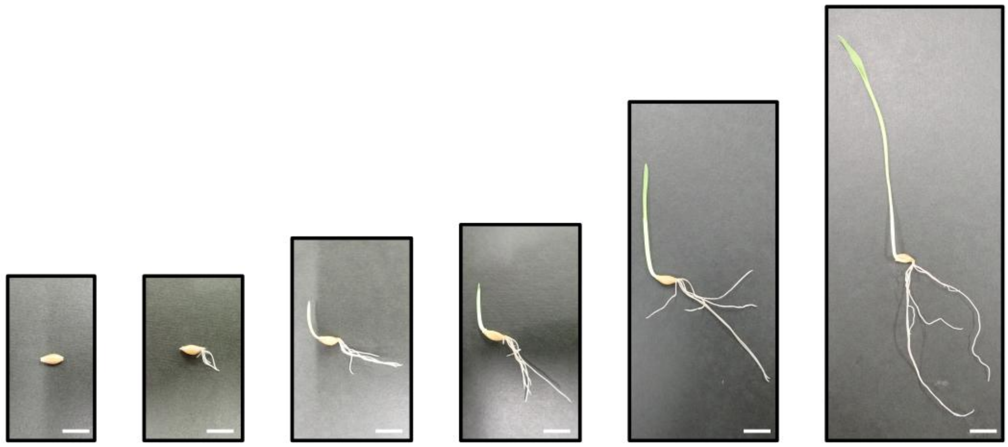

Supplementary Figure S1. Appearance of barley seedlings after seeding. Scale bars, 1 cm.

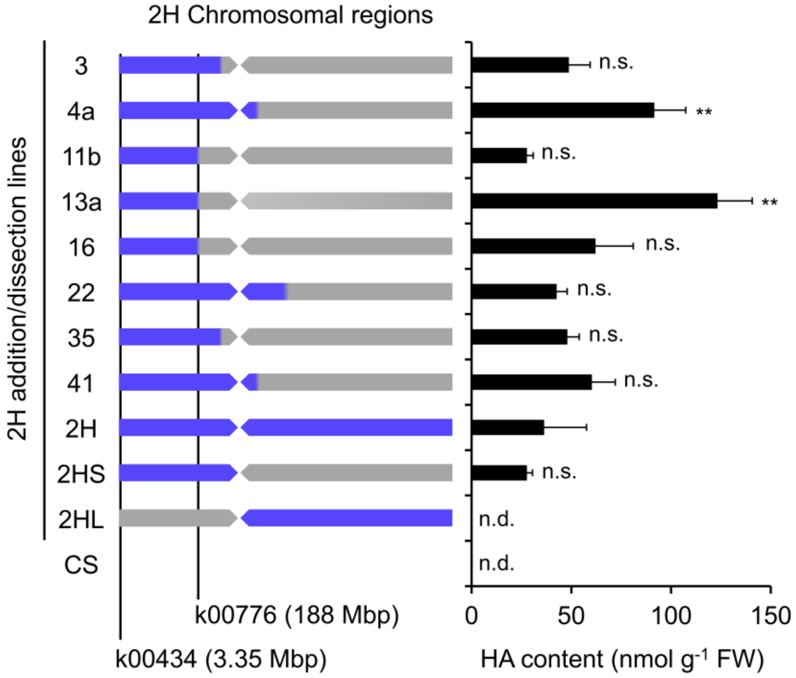

Supplementary Figure S2. Occurrence of hordatine A (HA) in wheat–barley 2H chromosome addition/dissection lines. Chromosomal region added to the wheat (cv. ‘Chinese Spring’; CS) genetic background and the HA content in 72-h-old shoots of each line. The 2H chromosomal region in each 2H addition/dissection line is presented in blue. Data are presented as the mean ± SD ($n = 3$). n.d.: not detected. The difference between 2H addition line and the other 2H addition/dissection lines was tested using Dunnett’s test. *: $P < 0.05$; **: $P < 0.01$ level. n.s., not significant ($P > 0.05$). Data are from Ube et al. (2023). See Supplementary Table S1 for EST markers.

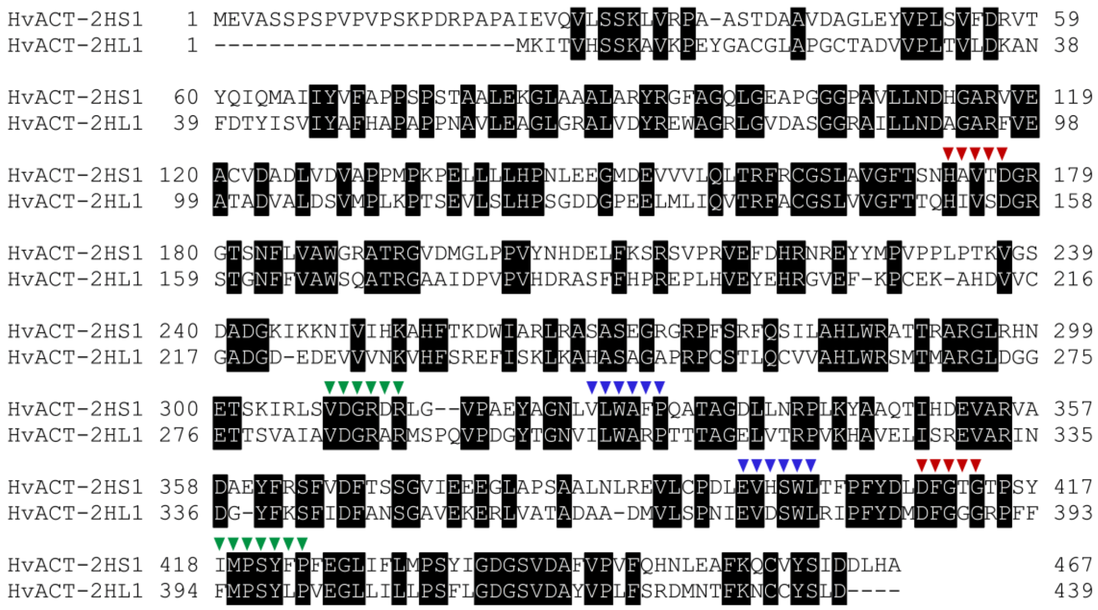

Supplementary Figure S3. Alignment of HvACT-2HS1 and HvACT-2HL1 amino acid sequences. Red arrowheads, conserved motifs in the BAHD acyltransferase superfamily (HXXXD and DFGWG); blue arrowheads, Clade IV-specific motifs (VLWAFP and EVDSWL); green arrowheads, Clade IVa-specific motifs (VDGRAR and FMPSYLP).

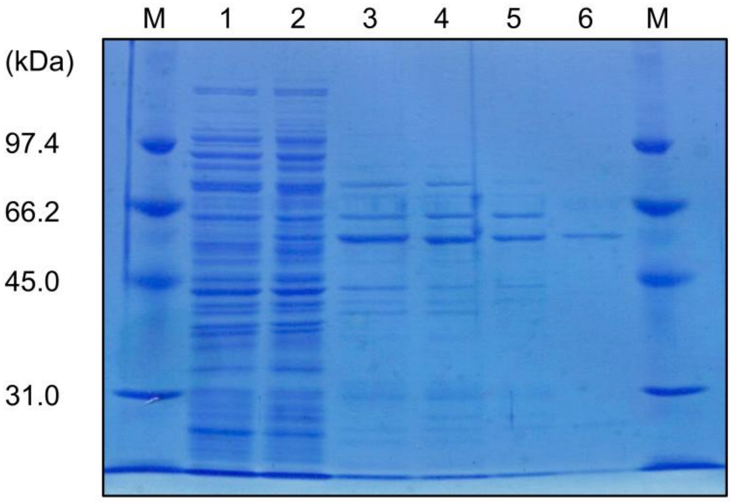

Supplementary Figure S4. Purification of recombinant HvACT-2HS1 expressed in *Escherichia coli*. Proteins from each purification step were separated by 10% SDS-PAGE and stained with CBB R-250. Lane M, molecular size markers; lane 1, crude *E. coli* extract before expression was induced; lane 2, crude *E. coli* extract after expression was induced; lane 3, eluate from first metal-affinity chromatography; lane 4, protein after thrombin digestion; lane 5, flowthrough from second metal-affinity chromatography; lane 6, DEAE-Toyopearl fraction.

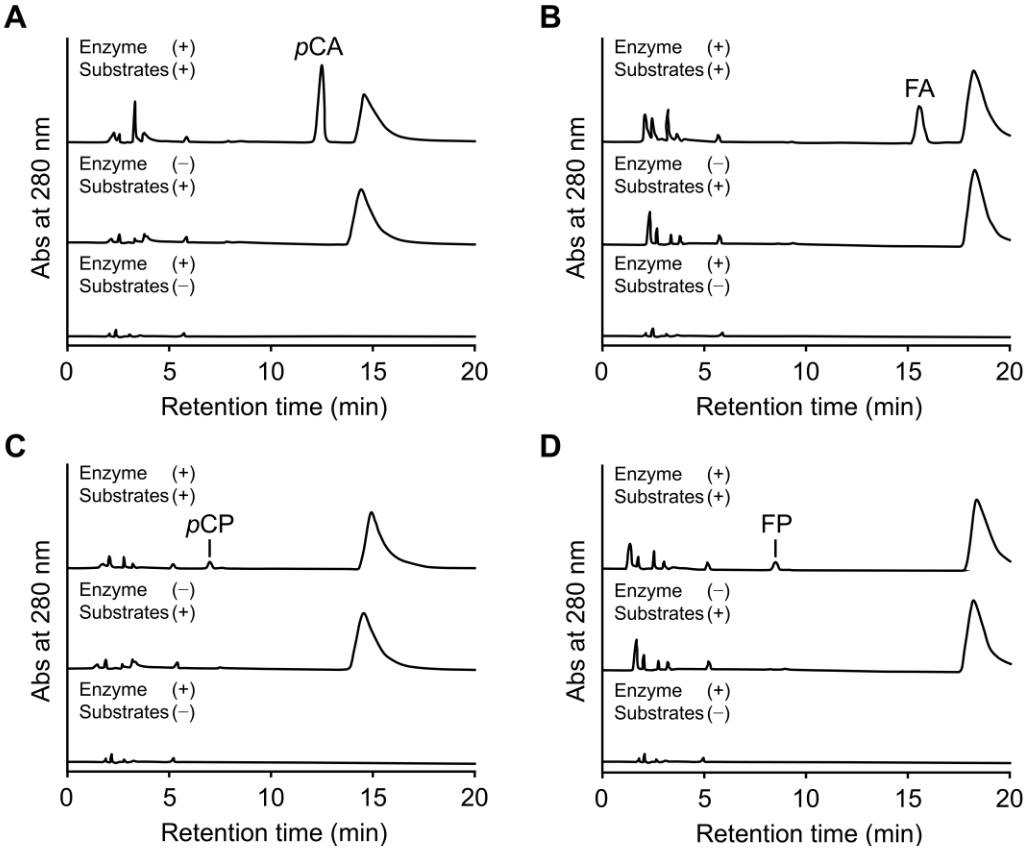

Supplementary Figure S5. Enzymatic activities of recombinant HvACT-2HS1 for the formation of *p*-coumaroylagmatine (*p*CA), feruloylagmatine (FA), *p*-coumaroylputrescine (*p*CP), and feruloylputrescine (FP). HPLC chromatograms of the reaction mixture for the formation of *p*CA (A), FA (B), *p*CP (C), and FP (D) in the presence (+) and absence (−) of the enzyme and two substrates (substrates for *p*CA formation, *p*-coumaroyl-CoA and agmatine; for FA formation, feruloyl-CoA and agmatine; for *p*CP formation, *p*-coumaroyl-CoA and putrescine; for FP formation, feruloyl-CoA and putrescine).

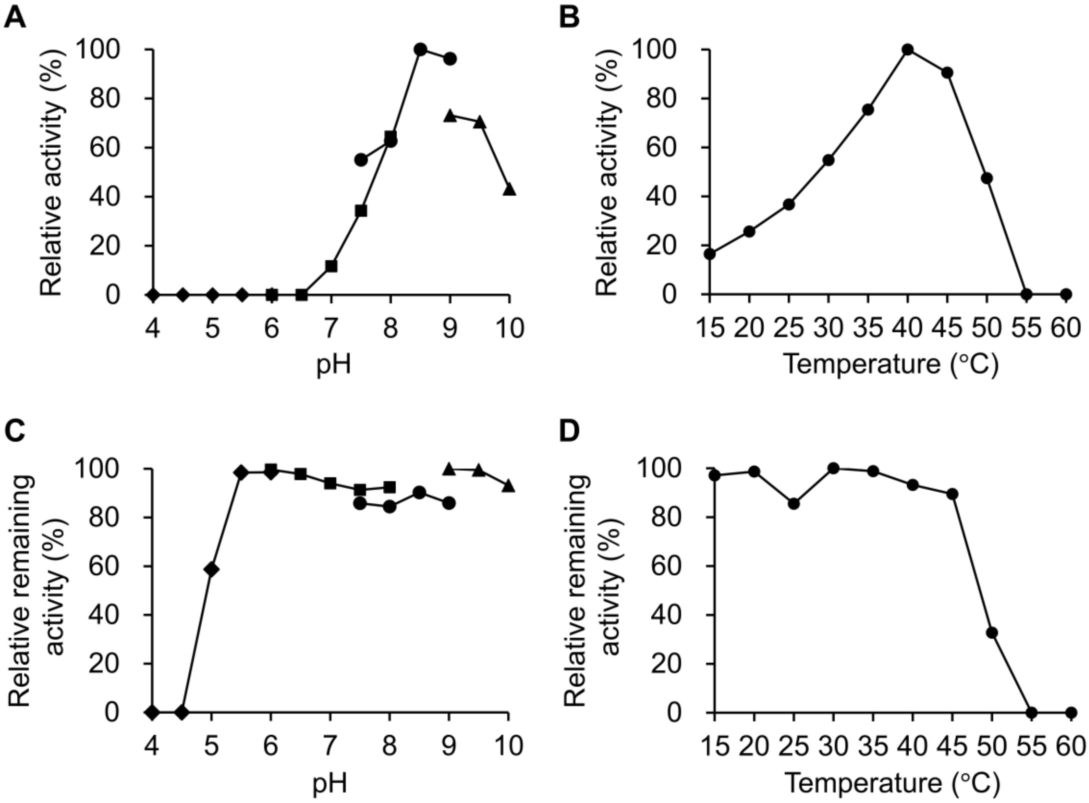

Supplementary Figure S6. Effects of pH and temperature on enzyme activity and stability of recombinant HvACT-2HS1. Optimal pH (A) and temperature (B) of the pCA-forming enzyme activity of recombinant HvACT-2HS1. The peak activities at pH 8.5 (A) and 40°C (B) were set to 100%. The enzyme reactions were performed under standard assay conditions at 40°C in 100 mM sodium acetate buffer (pH 4.0–6.0, diamonds), 100 mM KPi buffer (pH 6.0–8.0, squares), 100 mM Tris-HCl buffer (pH 7.5–9.0, circles), and 100 mM CHES-NaOH buffer (pH 9.0–10.0, triangles), or at 15–60°C in 100 mM Tris-HCl buffer (pH 8.5). To examine the effects of pH (C) and temperature (D) on enzyme stability, enzyme reactions were performed under standard assay conditions after treatment of the enzyme by incubation in the above-mentioned buffers (pH 4.0–10.0) for 30 min at 25°C, or in 100 mM KPi buffer (pH 7.5) at 15–60°C.

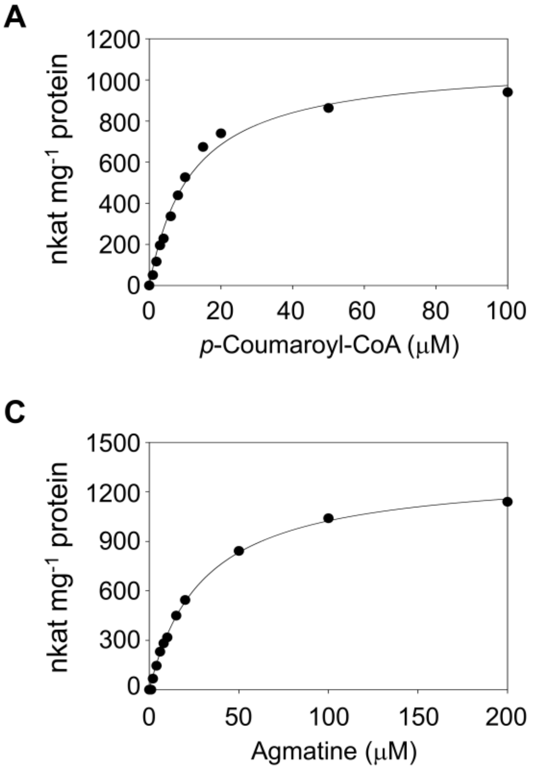

Supplementary Figure S7. Michaelis–Menten plots for reactions catalyzed by recombinant HvACT-2HS1 under optimal conditions. Formation of *p*CA (A) and FA (B) depending on the concentration of *p*-coumaroyl-CoA and feruloyl-CoA, respectively, in the presence of 100 μM agmatine. Formation of *p*CA (C) and *p*CP (D) depending on the concentration of agmatine and putrescine, respectively, in the presence of 100 μM *p*-coumaroyl-CoA. Enzymatic activity was calculated on the basis of the formation of the reaction product under standard assay conditions. Correlation coefficients (R^2) for the non-linear regression in (A), (B), (C), and (D) were 0.993, 0.974, 0.999, and 0.999, respectively. See Table 1 for kinetic parameters, which were calculated via non-linear regression-based fitting of the data to the Michaelis–Menten equation.

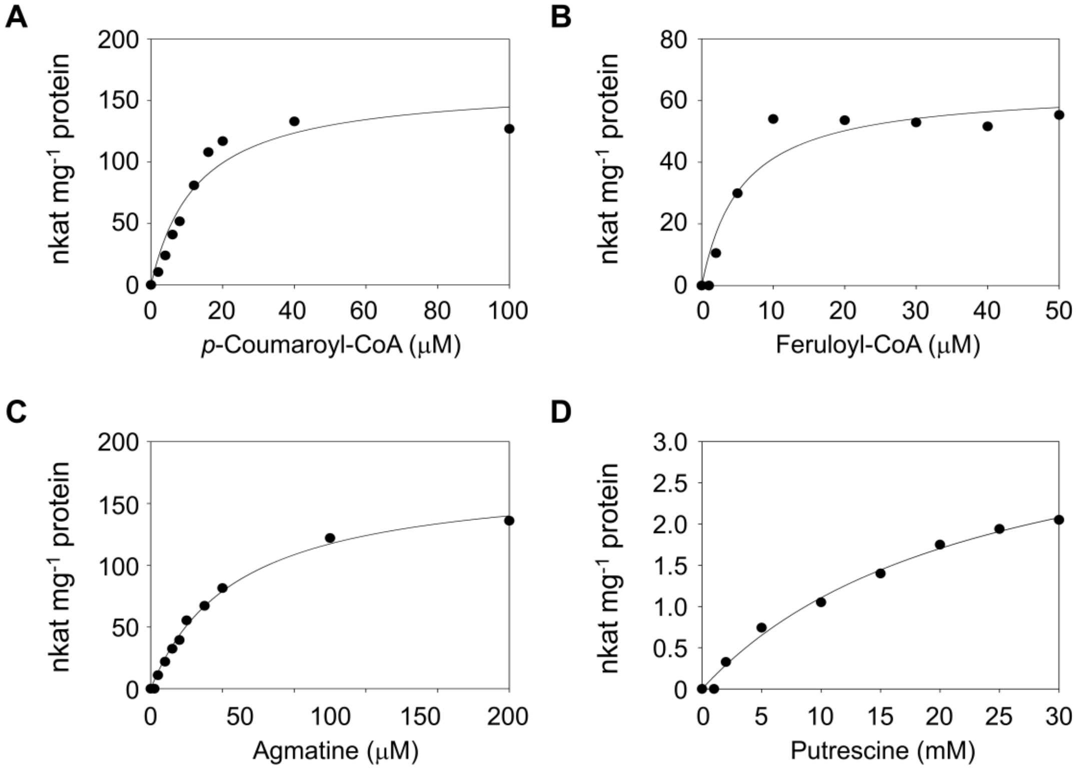

Supplementary Figure S8. Michaelis–Menten plots for reactions catalyzed by recombinant HvACT-2HS1 under the same conditions as those used for HvACT-2HL1. Formation of *p*CA (A) and FA (B) depending on the concentration of *p*-coumaroyl-CoA and feruloyl-CoA, respectively, in the presence of 100 μM agmatine. Formation of *p*CA (C) and *p*CP (D) depending on the concentration of agmatine and putrescine, respectively, in the presence of 100 μM *p*-coumaroyl-CoA. Enzymatic activity was calculated on the basis of the formation of the reaction product under the same conditions as those used for HvACT-2HL1 (Nomura et al. 2018). Correlation coefficients (R^2) for the non-linear regression in (A), (B), (C), and (D) were 0.964, 0.964, 0.997, and 0.996, respectively. See Table 2 for kinetic parameters, which were calculated via non-linear regression-based fitting of data to the Michaelis–Menten equation.

References

Carere J, Powell J, Fitzgerald T, Kazan K, Gardiner DM (2018) BdACT2a encodes an agmatine coumaroyl transferase required for pathogen defence in *Brachypodium distachyon*. *Physiol Mol Plant Pathol* 104: 69–76

Joshi GP, Nasuda S, Endo TR (2011) Dissection and cytological mapping of barley chromosome 2H in the genetic background of common wheat. *Genes Genet Syst* 86: 231–248

Nomura T, Ishizuka A, Kishida K, Islam AKMR, Endo TR, Iwamura H, Ishihara A (2007) Chromosome arm location of the genes for the biosynthesis of hordatines in barley. *Genes Genet Syst* 82: 455–464

Nomura T, Ogita S, Kato Y (2018) Rational metabolic-flow switching for the production of exogenous secondary metabolites in bamboo suspension cells. *Sci Rep* 8: 13203

Onkokesung N, Gaquerel E, Kotkar H, Kaur H, Baldwin IT, Galis I (2012) MYB8 controls inducible phenolamide levels by activating three novel hydroxycinnamoyl-coenzyme A: polyamine transferases in *Nicotiana attenuata*. *Plant Physiol* 158: 389–407

Peng M, Gao Y, Chen W, Wang W, Shen S, Shi J, Wang C, Zhang Y, Zou L, Wang S et al. (2016) Evolutionarily distinct BAHD *N*-acyltransferases are responsible for natural variation of aromatic amine conjugates in rice. *Plant Cell* 28: 1533–1550

Roumani M, Besseau S, Hehn A, Larbat R (2025) Functional characterization of a small gene family coding for putrescine hydroxycinnamoyltransferases, involved in phenolamide accumulation, in tomato. *Phytochemistry* 229: 114271

Tanabe K, Hojo Y, Shinya T, Galis I (2016) Molecular evidence for biochemical diversification of phenolamide biosynthesis in rice plants. *J Integr Plant Biol* 58: 903–913

Ube N, Yabuta Y, Tohnooka T, Ueno K, Taketa S, Ishihara A (2019) Biosynthesis of phenylamide phytoalexins in pathogen-infected barley. *Int J Mol Sci* 20: 5541

Ube N, Ishihara A, Yabuta Y, Taketa S, Kato Y, Nomura T (2023) Molecular identification of a laccase that catalyzes the oxidative coupling of a hydroxycinnamic acid amide for hordatine biosynthesis in barley. *Plant J* 115: 1037–1050

Yamane M, Takenoya M, Yajima S, Sue M (2021) Molecular and structural characterization of agmatine coumaroyltransferase in Triticeae, the key regulator of hydroxycinnamic acid amide accumulation. *Phytochemistry* 189: 112825
